# The Successful Use of Nitroglycerin for Uterine Hyperstimulation with Fetal Heart Rate Abnormality Caused by a Controlled-Release Dinoprostone Vaginal Delivery System (PROPESS): A Case Report

**DOI:** 10.3390/medicina57050478

**Published:** 2021-05-12

**Authors:** Sho Takakura, Hiroaki Tanaka, Naosuke Enomoto, Shintaro Maki, Tomoaki Ikeda

**Affiliations:** Department of Obstetrics and Gynecology, Mie University School of Medicine, 2-174 Edobashi, Tsu, Mie 514-8507, Japan; h_tanaka@med.miyazaki-u.ac.jp (H.T.); nao-e@clin.medic.mie-u.ac.jp (N.E.); mabochikin519@yahoo.co.jp (S.M.); t-ikeda@clin.medic.mie-u.ac.jp (T.I.)

**Keywords:** cervical ripening, dinoprostone, uterine hyperstimulation, fetal heart rate abnormality, nitroglycerin

## Abstract

The PROPESS, a controlled-release dinoprostone vaginal delivery system, is a pharmacological cervical ripening intervention and promotes cervical change causing uterine contraction. During insertion of the PROPESS, uterine hyperstimulation could occur and result in fetal heart rate (FHR) abnormality. We report a case of uterine hyperstimulation accompanied with FHR abnormality caused by the PROPESS in a pregnant woman. Postural change, oxygenation, fluid infusion, and the immediate PROPESS removal were ineffective to address the adverse event, so we administered nitroglycerin for acute uterine relaxation. The nitroglycerin resulted in uterine relaxation, and the FHR abnormality was resolved immediately, thereby preventing an emergency cesarean section. Therefore, nitroglycerin could be considered an effective option for uterine hyperstimulation accompanied with FHR abnormality caused by the PROPESS.

## 1. Introduction

Labor induction is a common obstetric intervention. In pregnant women who need it and have insufficient cervical ripening at the start of induction, the risk of undergoing a cesarean section is increased, and therefore, a cervical ripening intervention is required [1]. A controlled-release dinoprostone vaginal delivery system (PROPESS) that is a pharmacological cervical ripening intervention has already been used worldwide [2,3]. Although the PROPESS had not been used in Japan, a Japanese clinical study on its use was conducted [4], and the PROPESS was approved by the Ministry of Health, Labour, and Welfare in January 2020. The PROPESS has a similar success rate of labor induction with a mechanical cervical ripening intervention [5,6]. The PROPESS would become common in Japan because of less pain associated with insertion and a lower risk of intrauterine infection than the mechanical cervical ripening intervention [7]. However, the PROPESS promotes cervical change with causing uterine contraction compared to the mechanical cervical ripening intervention, and uterine hyperstimulation could occur [8]. Moreover, uterine hyperstimulation occasionally results in fetal heart rate (FHR) abnormality. Therefore, a quick and appropriate response to uterine hyperstimulation accompanied with FHR abnormality during the insertion of the PROPESS is required.

Here, we report a case of uterine hyperstimulation accompanied with FHR abnormality caused by the PROPESS in a pregnant woman, who was administered with nitroglycerin, which resulted in the prevention of an emergency cesarean section.

## 2. Case

**Figure 1:**
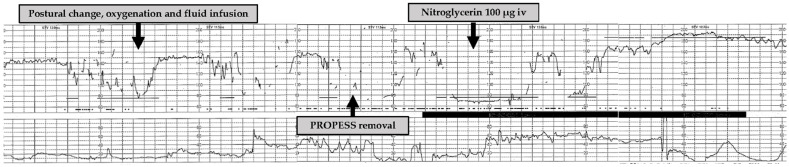
Cardiotocogram at the occurrence of uterine hyperstimulation accompanied with fetal heart rate abnormality caused by the PROPESS.

A 32-year-old woman (gravida 4, para 2) became pregnant spontaneously and was referred to our institution at 11 weeks and 4 days of gestation because her previous pregnancy had resulted in intrauterine fetal death (IUFD) at 40 weeks and 4 days of gestation. The cause of IUFD was unknown. She was hospitalized at 37 weeks and 4 days of gestation and planned to undergo labor induction because of her previous pregnancy history. Transabdominal ultrasonography showed a cephalic-presenting fetus weighing 2918 g (−0.2 standard deviation) surrounded by normal amniotic fluid volume, and her Bishop score was 1 at 39 weeks and 0 days of gestation. A cervical ripening intervention was required because of insufficient cervical ripening. After obtaining informed consent, we used the PROPESS to promote her cervical ripening. At 30 min after insertion of the PROPESS, uterine hyperstimulation and recurrent prolonged deceleration occurred (Figure 1). Uterine contractions were monitored externally using a tocodynamometer and showed inadequate registration of contractions, and uterine hyperstimulation was determined by the use of palpation. We performed postural change, oxygenation, and fluid infusion, and the PROPESS was immediately removed. They were ineffective to address the adverse event, and therefore, we administered 100-μg nitroglycerin intravenously for acute uterine relaxation. The FHR abnormality was resolved immediately, and uterine relaxation was achieved, thereby preventing an emergency Cesarean section. At 4 h and 15 min after the insertion of the PROPESS, a 2832-g baby was delivered with Apgar scores of 8 and 9 at 1 min and 5 min after birth, respectively. The umbilical arterial pH was 7.328.

## 3. Discussion

This is the first case report of a successful use of nitroglycerin for uterine hyperstimulation accompanied with FHR abnormality caused by the PROPESS.

Prostaglandin E_2_ exerts an oxytocic effect on the myometrium, which increases uterine tone and tends to decrease uteroplacental perfusion [9]. Uterine hyperstimulation is a common side effect of prostaglandin preparations used for labor induction [8]. Additionally, Chen et al. reported that the use of prostaglandin to induce labor was associated with a higher rate of uterine hyperstimulation accompanied with FHR changes than the mechanical cervical ripening intervention [5]. Immediate removal of the PROPESS is possible to prevent it from releasing more dinoprostone if uterine hyperstimulation occurs. However, in this case, despite the postural change, oxygenation, fluid infusion, and immediate removal of the PROPESS when the uterine hyperstimulation accompanied with FHR abnormality had occurred, the adverse event was not improved easily. In such a situation, the administration of nitroglycerin for acute uterine relaxation may be effective.

Nitroglycerin relaxes the smooth muscle. By intravenous administration, it acts within a few seconds and has a half-life of only a few minutes [10]. Axemo et al. reported that after injection of 100–200-μg nitroglycerin to 32 pregnant women during a cesarean section when uterine relaxation was urgently needed and to 22 pregnant women after vaginal delivery for facilitation of the manual removal of retained placentas, uterine relaxation was achieved within 45–60 s, and it lasted no longer than 2 min [11]. Nitroglycerin for uterine relaxation is fast-acting and lasts for a short duration, making it highly tunable. Therefore, the administration of nitroglycerin against uterine hyperstimulation accompanied with FHR abnormality may lead to improving placental blood flow and fetal oxygenation by rapidly relaxing the uterine smooth muscle. We believe that the most effective and immediate way against uterine hyperstimulation accompanied with FHR abnormality is the administration of nitroglycerin, not lateral decubitus positioning and intravenous fluid bolus. Maternal oxygen administration has no impact on improving neonatal outcomes [12]. Regarding the side effects of nitroglycerin, occasional mild hypotension has been noticed, but no increased heart rate in mothers [11].

The systematic review by Leathersich et al. focusing on acute tocolysis for uterine tachysystole or suspected fetal distress during labor, showed insufficient evidence to determine the effects of tocolytics, including nitroglycerin, beta-mimetic agent, oxytocin receptor antagonist, and so on [13]. The use of beta-mimetic agents consistently provokes tachycardia and hypotension and has also been associated with maternal cardiovascular instability and pulmonary edema [11]. This is one of the reasons why we choose nitroglycerin, not beta-mimetic agents other than the rapid onset of nitroglycerin effect and short half-life.

Further consideration will be needed about the use of nitric oxide (NO) donors for preventing fetal decompensation and reducing the risk of fetal distress. Turner et al. have recently reported that maternal oral sildenafil citrate (SC), a phosphodiesterase type 5 inhibitor and potent vasodilator mediated through increased NO bioavailability, given during labor substantially reduced the risk of cesarean section or instrumental delivery for presumed intrapartum fetal compromise [14]. They also have shown that it may be possible to identify fetuses that would most benefit from intrapartum maternal SC-based upon fetoplacental blood flow indices [15]. In the future, it might be good to give NO donors during labor for cases suspected placental insufficiencies, such as fetal growth restriction, and the use of PROPESS.

In conclusion, nitroglycerin could be considered an effective option for uterine hyperstimulation accompanied with FHR abnormality caused by the PROPESS.
